# Restoring colistin sensitivity in colistin-resistant gram-negative bacteria: combinatorial use of α-terpineol and colistin

**DOI:** 10.1128/spectrum.00825-25

**Published:** 2025-09-30

**Authors:** Yuhan Yang, Panjie Hu, Yanchun Gong, Zeyong Zhong, Yichi Zhang, Fan Ye, Tieli Zhou, Jianming Cao, Zhongyong Wang

**Affiliations:** 1 School of Laboratory Medicine and Life Science, Wenzhou Medical University 26453, Wenzhou, China; 2 Department of Clinical Laboratory, The First Affiliated Hospital of Wenzhou Medical University 89657, Wenzhou, China; Yan'an University, Yan'an, Shaanxi, China

**Keywords:** gram-negative bacteria, colistin resistance, α-terpineol, synergistic antimicrobial effects, biofilm

## Abstract

Multidrug-resistant gram-negative bacteria (GNB) have posed a serious public health hazard worldwide. Colistin (COL), the ultimate therapeutic option for treating GNB infections, has had escalating resistance rates concomitant with increased utilization. In this study, we attempted to increase the sensitivity to COL using a plant extract, α-terpineol (α-TP). Checkerboard and time-kill assays indicated that the combination of COL and α-TP exhibited pronounced synergistic antimicrobial effects against colistin-resistant (COL-R) GNB *in vitro*. Biofilm assessments revealed that COL combined with α-TP effectively inhibited biofilm development and eliminated established biofilms. Scanning electron microscopy indicated substantial disruption of bacterial cell membranes after 6 h of combined treatment. Additionally, cytotoxicity and erythrocyte hemolysis assays confirmed that α-TP maintained a degree of safety at synergistic concentrations. Mechanistically, the combination of COL and α-TP promoted the accumulation of reactive oxygen species and enhanced the permeability of both the inner and outer bacterial membranes. This study demonstrated that α-TP can restore the sensitivity of COL-R GNB to COL when used in combination.

**IMPORTANCE:**

Colistin (COL) has emerged as a last-line therapeutic agent for gram-negative bacteria (GNB) infections. However, widespread antibiotic use has led to an alarming increase in the number of colistin-resistant (COL-R) strains. In this study, we demonstrate that combining COL with a plant-derived extract could effectively restore the susceptibility of COL-R GNB. A possible treatment plan for COL-R GNB is presented.

## INTRODUCTION

The prevalence of multidrug-resistant (MDR) gram-negative bacteria (GNB) is increasing sharply (1). Global observations of antimicrobial resistance and escalating prevalence of infections caused by multiple pathogens are concerning. Due to this resistance, projected global economic repercussions are expected to exceed $105 billion each year (2). Irrational use and misuse of antibiotics have been identified as primary contributors (3). Researchers have suggested that limiting antibiotic use can help mitigate resistance rates. However, reducing the optimal dose of antibiotics may compromise treatment efficacy (4).

Colistin (COL) was discontinued owing to its nephrotoxicity and neurotoxicity. However, commonly used antibacterial drugs are ineffective with an increase in MDR GNB infections and evolution of bacterial resistance, and COL has been reintroduced into clinical settings (5). COL is regarded as the last option for treating GNB infections. Regrettably, chromosomal mutations and the spread of plasmids with resistance genes are responsible for the increase in COL resistance (6). The contemporary mechanisms of resistance to COL primarily involve structural modifications of lipopolysaccharides (LPSs), regulation of two-component systems, overexpression of efflux pumps, and widespread dissemination of drug-resistant plasmids such as mcr-1 in clinical environments (7, 8). To effectively address MDR GNB and mitigate the adverse effects associated with COL use in clinical practice, it is critical to identify appropriate therapeutic strategies to enhance the antimicrobial efficacy of COL.

α-Terpineol (α-TP) is a flavoring agent that naturally occurs in various plant species and is widely used in the formulation of perfumes, fragrances, food items, and cosmetics (9, 10). In the realm of medicine, α-TP exhibits notable pharmacological properties, including anti-inflammatory, antipruritic, and analgesic effects (9–11). α-TP also possesses antitumor potential, making it a candidate for anticancer drug development (12). Additionally, α-TP has demonstrated antibacterial activity against *Escherichia coli* O157:H7, *Salmonella typhi*, and *Staphylococcus aureus* (13). However, to the best of our knowledge, the combined use of α-TP and COL to treat GNB has not previously been reported.

Our investigation demonstrated that α-TP improved the susceptibility of colistin-resistant (COL-R) GNB to COL. Combined therapy enhanced the antibacterial and antibiofilm properties of COL-R GNB *in vitro* and *in vivo*, indicating a viable clinical treatment strategy for COL-R GNB infection.

## RESULTS

### Antimicrobial susceptibility tests

Most of the strains used in this study were MDR (25/32). Minimum inhibitory concentrations (MICs) of commonly used antibiotics in clinical settings are shown in Table S1. These strains had MICs of ≥4 µg/mL for COL, and all MICs were ≥512 µg/mL for α-TP, which indicated that α-TP had poor antibacterial activity against the tested COL-R GNB strains.

### Checkerboard assay

When combined with α-TP, MICs of COL decreased by 4- to 2,048-fold, and fractional inhibitory concentration index (FICI) values ranged from 0.046875 to 0.5 (Table 1). This indicated that COL in combination with α-TP had a synergistic antimicrobial effect on all tested strains (FICI ≤ 0.5). In the presence of α-TP, all isolates became sensitive to COL again. Meanwhile, we also performed checkerboard assays to evaluate the combined effects of α-TP with eight colistin-sensitive (COL-S) bacterial strains. FICI values ranged from 0.1875 to 0.375 (Table S2).

**TABLE 1:** FICI values for colistin/α-terpineol combinations against COL-R GNB^*a*^

Species	Strain	Monotherapy MIC (μg/mL)		Combination MIC (μg/mL)		FICI	Interpretation
		Colistin	α-Terpineol	Colistin	α-Terpineol		
*Escherichia coli*	DC90	4	1,024	1	128	0.375	Synergistic
	DC3599	8	1,024	2	256	0.5	Synergistic
	DC3737	4	1,024	1	128	0.375	Synergistic
	DC3846	4	1,024	1	16	0.265625	Synergistic
	DC5286	4	1,024	1	256	0.5	Synergistic
	DC19144	8	1,024	2	128	0.375	Synergistic
	DC19526	4	1,024	1	128	0.375	Synergistic
	DC19829	8	1,024	0.125	128	0.140625	Synergistic
*Klebsiella pneumoniae*	FK169	≥256	1,024	0.125	256	≤0.250488	Synergistic
	FK1913	≥256	≥2,048	0.125	256	≤0.125488	Synergistic
	FK3994	64	1,024	0.5	256	0.257813	Synergistic
	FK6556	8	1,024	0.5	256	0.3125	Synergistic
	FK6663	4	≥2,048	1	64	≤0.28125	Synergistic
	FK6696	128	1,024	0.25	256	0.251953	Synergistic
	FK11237	4	1,024	0.125	128	0.15625	Synergistic
	FK12716	16	1,024	0.5	64	0.09375	Synergistic
	FK12771	64	1,024	0.25	256	0.253906	Synergistic
*Acinetobacter baumannii*	BM1342	4	1,024	1	128	0.375	Synergistic
	BM1412	4	1,024	1	16	0.265625	Synergistic
	BM2431	4	1,024	0.5	256	0.375	Synergistic
	BM7477	128	1,024	0.5	256	0.253906	Synergistic
	BM7970	128	512	1	128	0.257813	Synergistic
	BM7994	16	1,024	1	128	0.1875	Synergistic
	BM8014	16	1,024	0.5	128	0.15625	Synergistic
*Pseudomonas aeruginosa*	TL1671	4	≥2,048	0.5	256	≤0.25	Synergistic
	TL7333	16	≥2,048	1	128	≤0.125	Synergistic
	TL7440	16	≥2,048	0.5	128	≤0.09375	Synergistic
	TL7505	8	≥2,048	0.25	128	≤0.09375	Synergistic
	TL7548	16	≥2,048	2	128	≤0.1875	Synergistic
	TL7733	8	≥2,048	1	128	≤0.1875	Synergistic
	TL7929	32	≥2,048	0.5	64	≤0.046875	Synergistic
	TL8126	32	1,024	0.5	256	0.265625	Synergistic

^*a*^ FICI, fractional inhibitory concentration index.

### Time-kill assays

**Fig 1:**
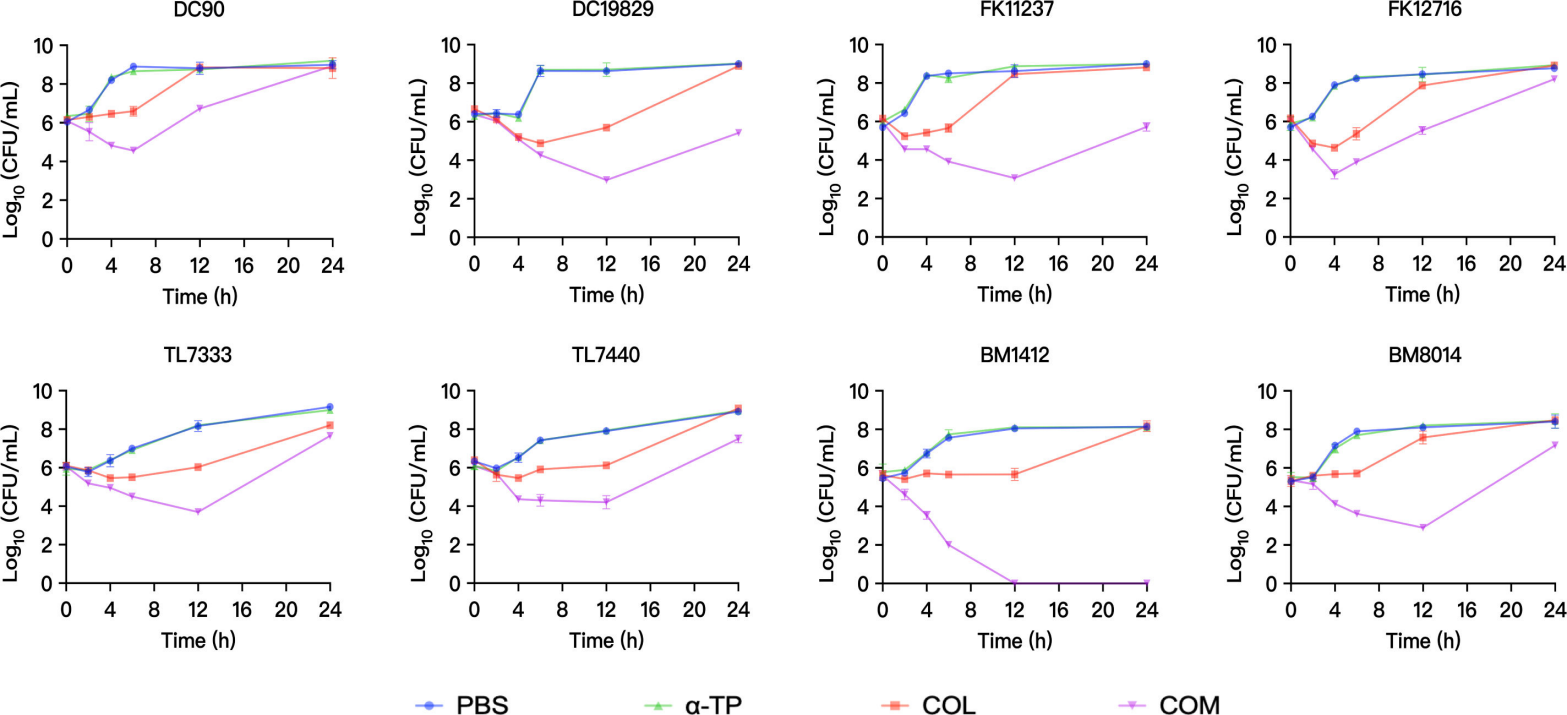
Time-kill assay of COL and α-TP alone and in combination against COL-R GNB. COM, combination group; DC, *Escherichia coli*; FK, *Klebsiella pneumoniae*; TL, *Pseudomonas aeruginosa*; and BM, *Acinetobacter baumannii*.

To investigate the combined dynamic bacterial impact of COL and α-TP, 8 strains were randomly selected from 32 strains for time-kill assays (Fig. 1). The concentrations of the two compounds were selected based on checkerboard assay results. Based on the experimental results, compared with monotherapy, the combination therapy achieved a ≥2 log_10_ colony-forming unit (CFU/mL) reduction against most bacterial strains within 6–12 h of treatment. However, for most strains, complete bacterial eradication was not attained even at 2× FICI combination concentrations, with bacterial regrowth observed at 24 h. Except for BM1412, no bacterial growth was detected after 12 h of combination treatment.

### Impact on biofilm formation and eradication

**Fig 2:**
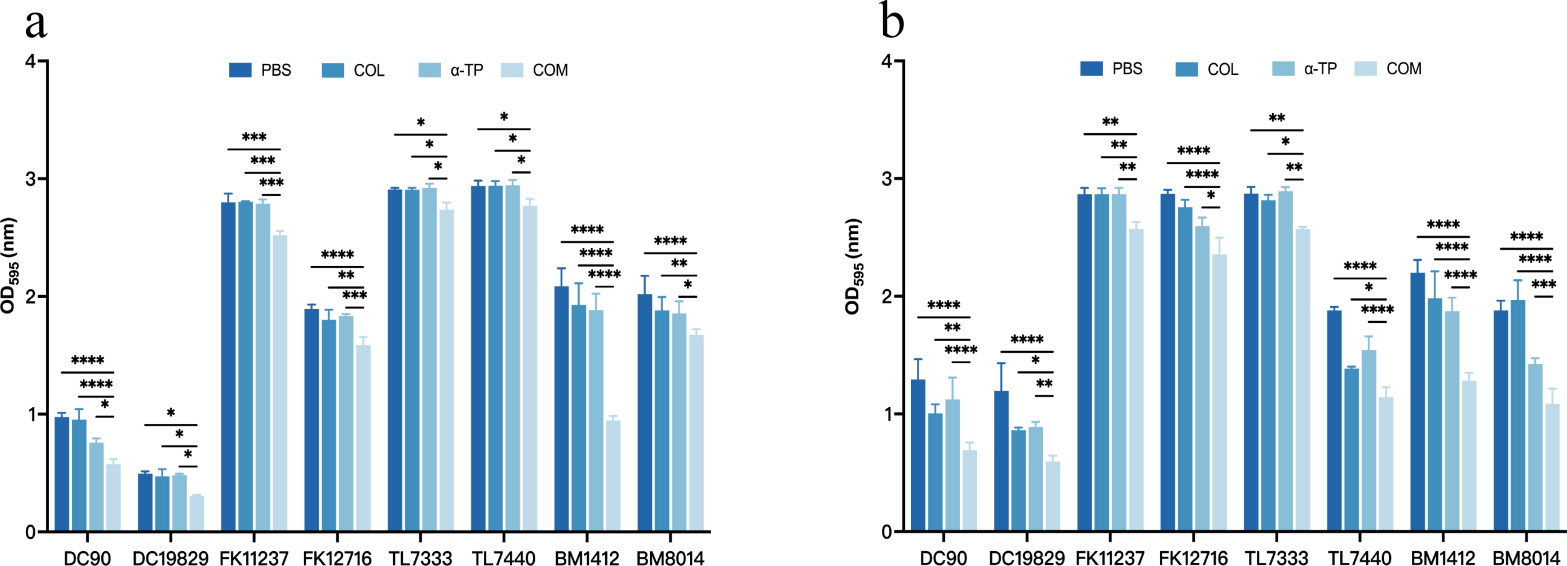
CV staining assay was performed to detect the antibiofilm effects. (**a**) Biofilm inhibition assay. (**b**) Mature biofilm elimination assay. COM, combination group; DC, *Escherichia coli*; FK, *Klebsiella pneumoniae*; TL, *Pseudomonas aeruginosa*; and BM, *Acinetobacter baumannii*. *P < 0.05; **P < 0.01; ***P < 0.001; and ****P < 0.0001.

**Fig 3:**
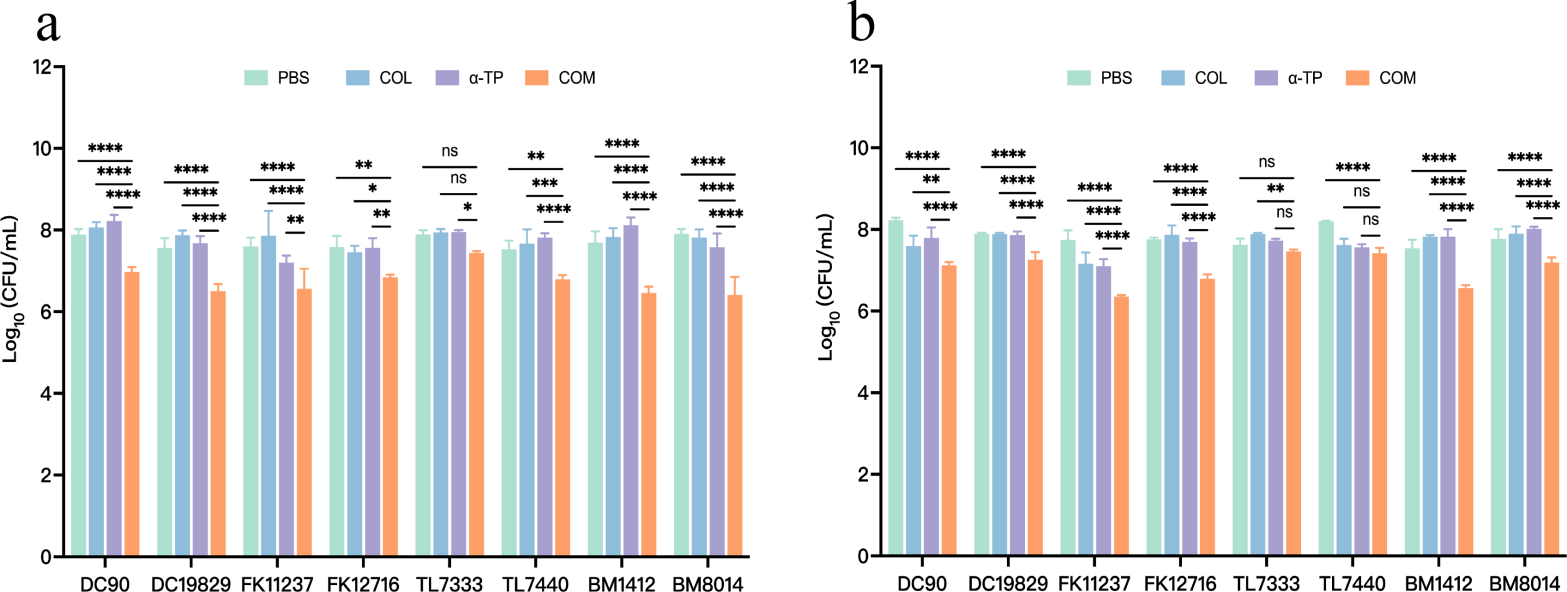
Count of live bacteria in biofilms. (**a**) Biofilm formation inhibition assay. (**b**) Mature biofilm eradication assay. COM, combination group; DC, *Escherichia coli*; FK, *Klebsiella pneumoniae*; TL, *Pseudomonas aeruginosa*; and BM, *Acinetobacter baumannii*. *P < 0.05; **P < 0.01; ***P < 0.001; ****P < 0.0001; and ns, *P* > 0.05.

**Fig 4:**
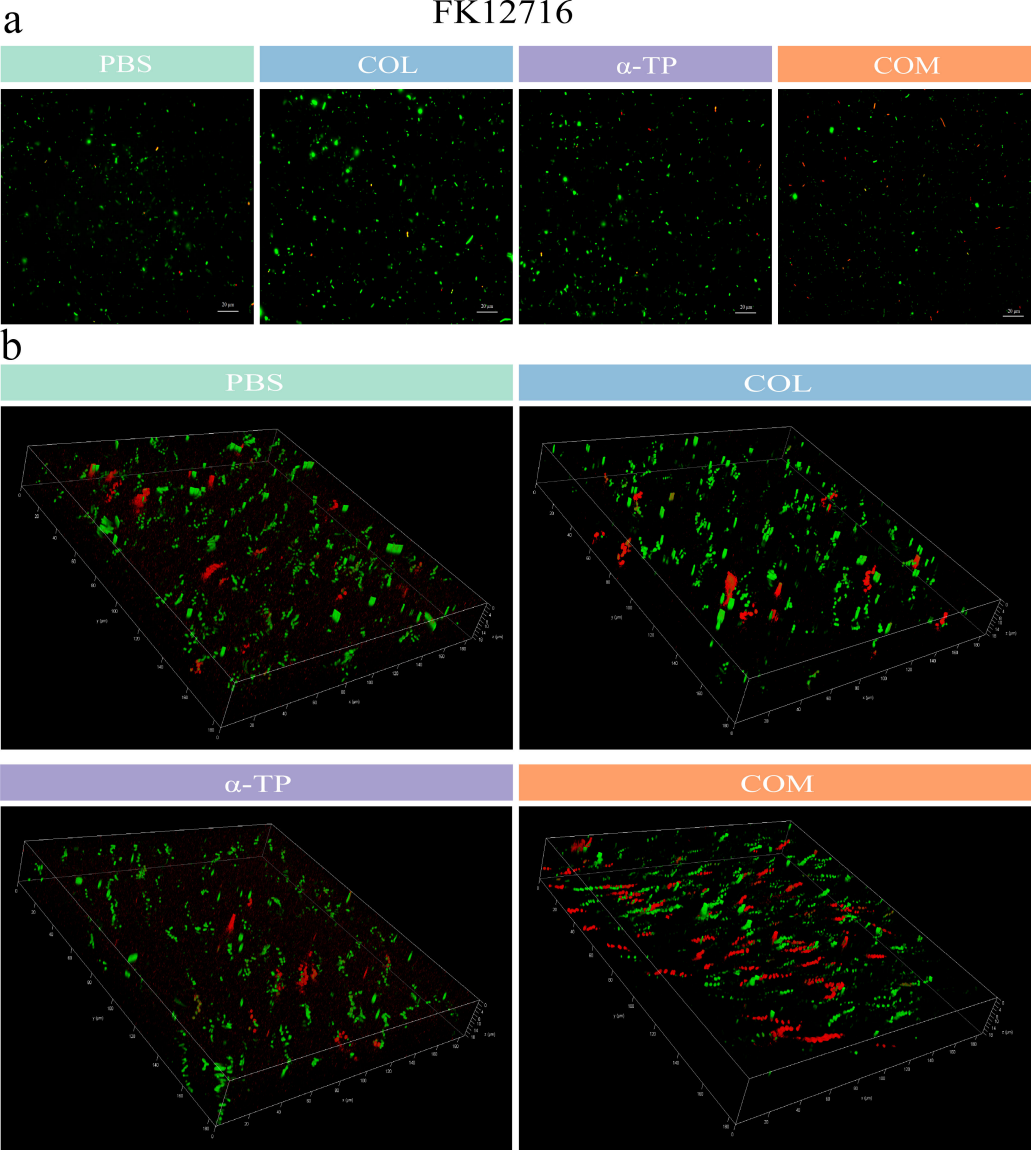
CLSM was employed to visualize live/dead bacterial distributions within biofilms. (**a**) Two-dimensional fluorescence dual staining. (**b**) Three-dimensional panoramic scanning of the biofilm. COM, combination group; FK, *Klebsiella pneumoniae*.

Eight strains (DC19829, DC90, FK11237, FK12716, TL7440, TL7333, BM8014, and BM1412) were randomly selected from the 32 tested strains. Crystal violet staining was used to investigate the inhibitory effects of COL combined with α-TP on biofilms and their capacity to remove established biofilms. COL combined with α-TP had a significant inhibitory effect on biofilm formation compared with that in other groups (*P* < 0.05) and also had an expressive eradicative effect on formed biofilm (*P* < 0.05) (Fig. 2). We quantified the viable bacteria in the biofilms. In both the biofilm formation inhibition and mature biofilm eradication experiments, the bacterial counts in the biofilms formed by most strains were reduced (Fig. 3). Confocal laser scanning microscopy (CLSM) results also demonstrated a reduction in viable bacterial counts within the biofilms after combination treatment with FK12716 (Fig. 4).

### Safety tests *in vitro*

**Fig 5:**
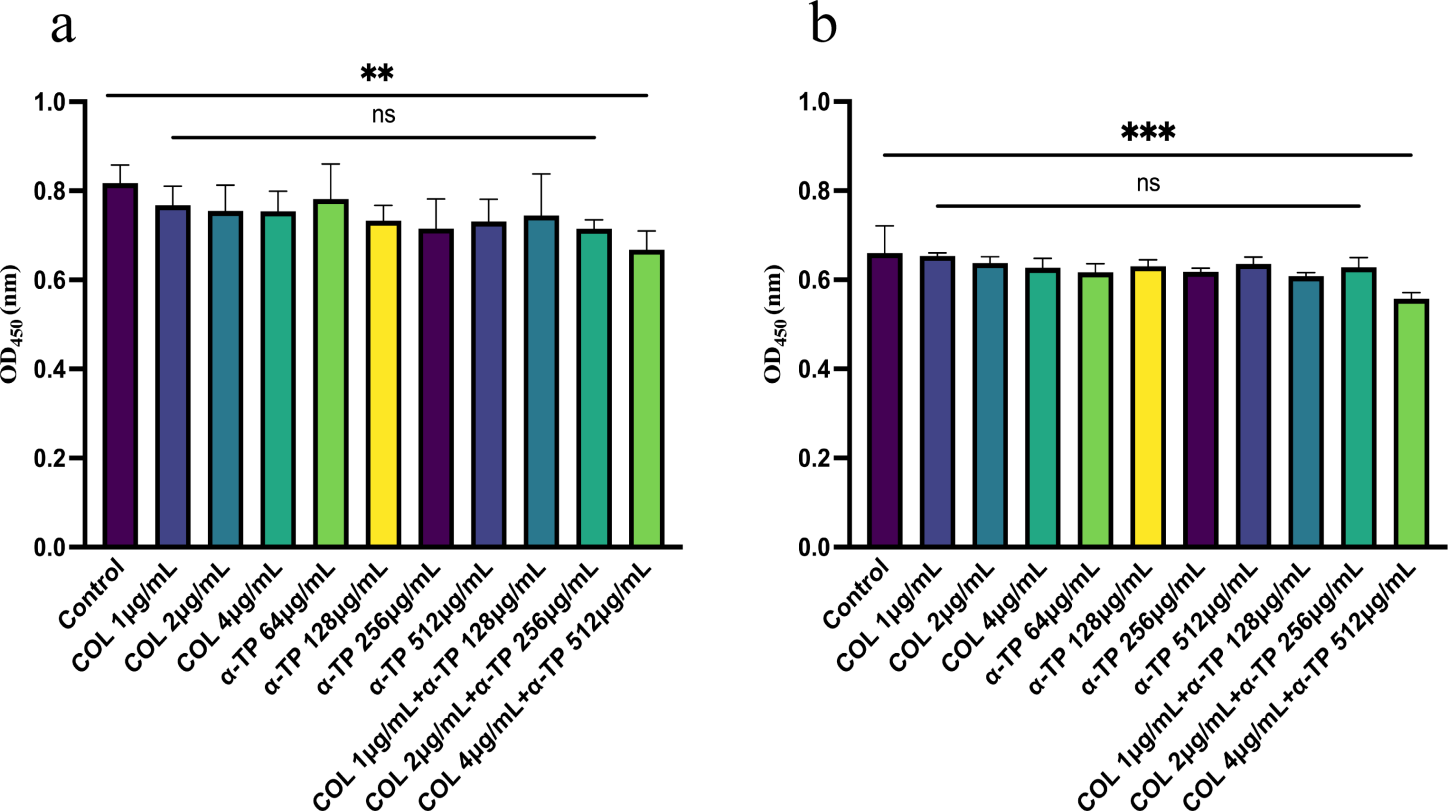
Toxicity of COL, α-TP, and combination groups on (**a**) RAW264.7 cells and (**b**) HEK-293T cells. **P < 0.01; ***P < 0.001; and ns, *P* > 0.05.

RAW264.7 and HEK-293T cells were used to investigate the potential cytotoxicity of α-TP and α-TP combined with COL (Fig. 5). No cytotoxicity was evident in the treatment groups relative to the control group when the α-TP concentration increased to 512 µg/mL.

**Fig 6:**
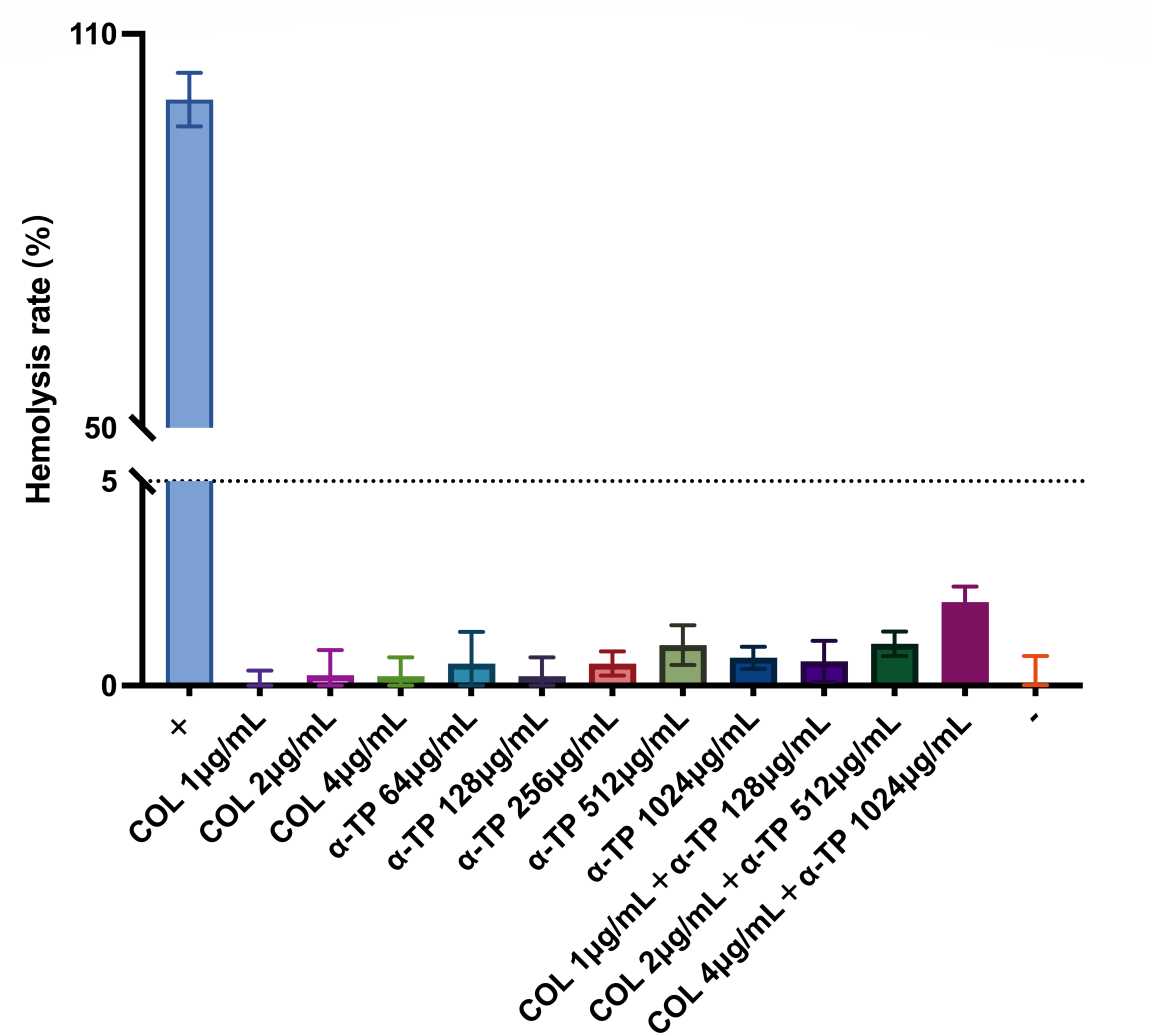
Hemolytic rate of red blood cells after treatment with COL, α-TP, and combination groups on mice erythrocytes.

Venous blood from mice was used for erythrocyte hemolysis experiments (Fig. 6). The results showed that erythrocyte hemolysis did not occur even after treatment with high α-TP concentrations (1,024 µg/mL) alone or in combination, suggesting that the combination regimen in this study has an acceptable *in vitro* safety profile.

### Safety tests *in vivo*

**Fig 7:**
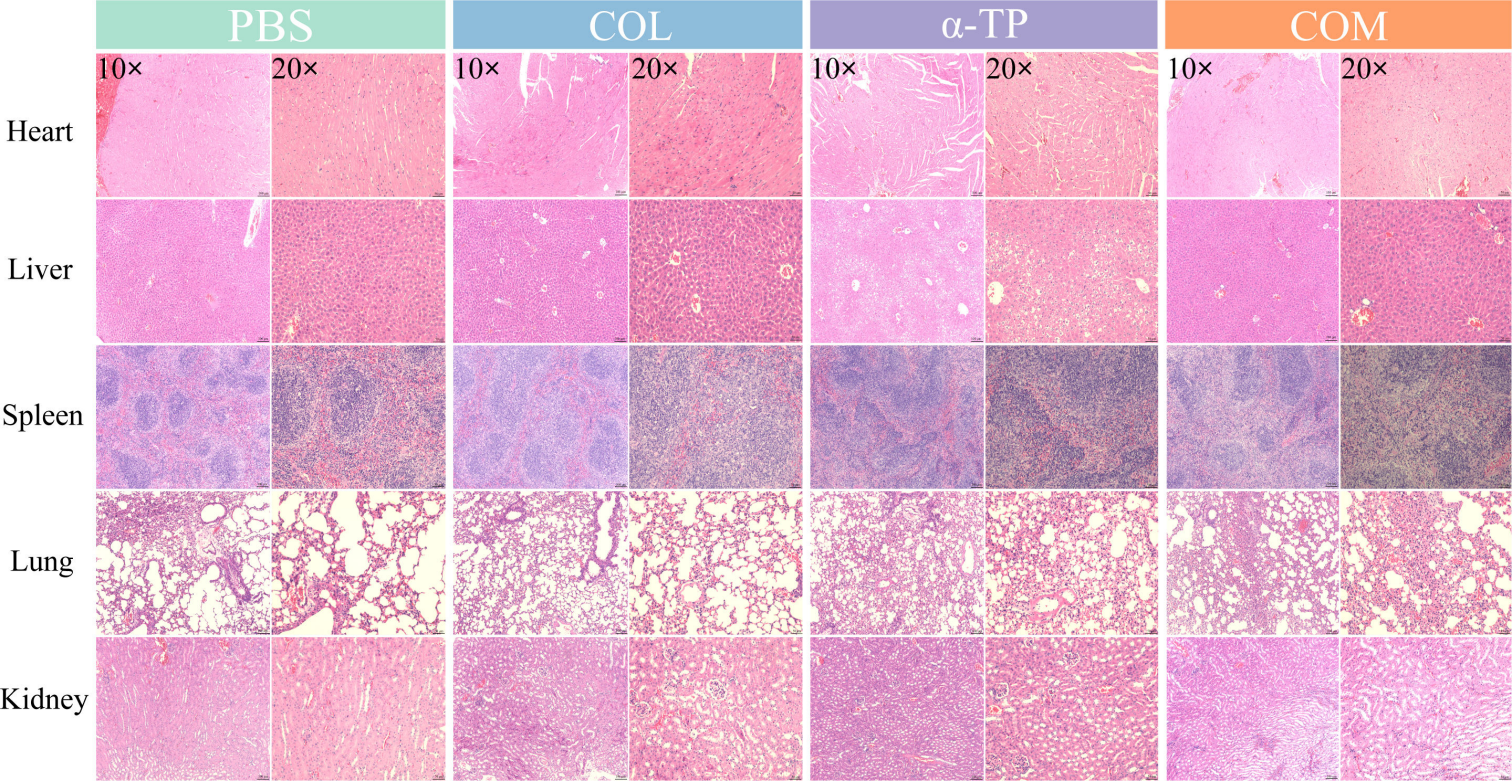
Histological sections of heart, liver, spleen, lung, and kidney tissues from compound-injected mice. COM, combination group.

To preliminarily evaluate the *in vivo* safety of the compounds, we observed hematoxylin‒eosin (H&E) staining of tissue sections from the heart, liver, spleen, lungs, and kidneys of mice (Fig. 7). The experimental results showed that after seven consecutive days of intraperitoneal compound administration, the histological features of these organs in treated mice were comparable to those in the control group, with no signs of inflammation, indicating a healthy state. These preliminary findings confirmed that both monotherapy and combination therapy exhibited acceptable safety profiles in mice.

### Mechanisms for drug synergy

**Fig 8:**
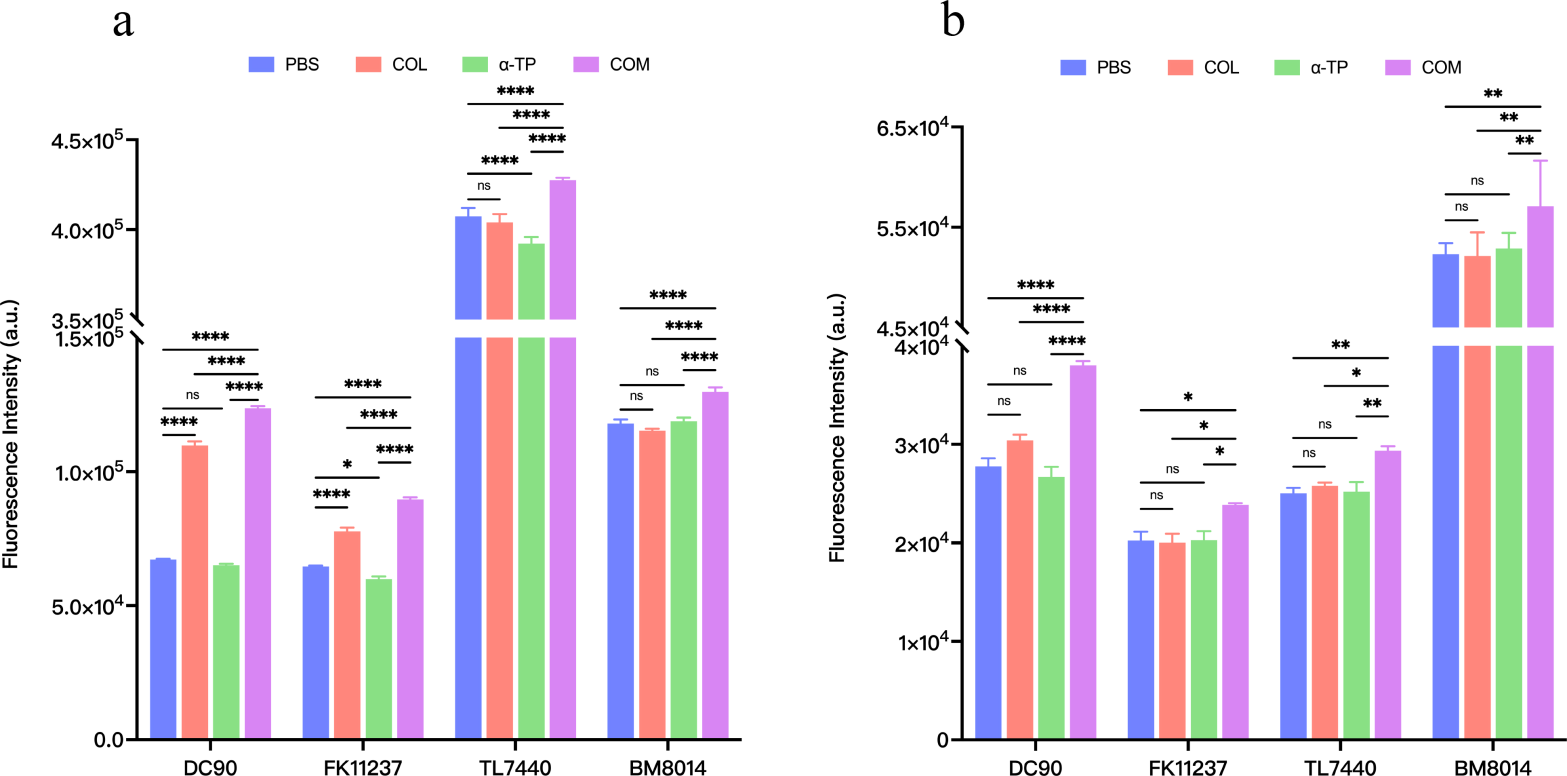
Investigation of synergistic antibacterial mechanisms. (**a**) Reactive oxygen species and (**b**) superoxide anion production levels of DC90, FK11237, TL7440, and BM8014 after different treatments. COM, combination group; DC, *Escherichia coli*; FK, *Klebsiella pneumoniae*; TL, *Pseudomonas aeruginosa*; and BM, *Acinetobacter baumannii*. *P < 0.05; **P < 0.01; ****P < 0.0001; and ns, *P* > 0.05.

**Fig 9:**
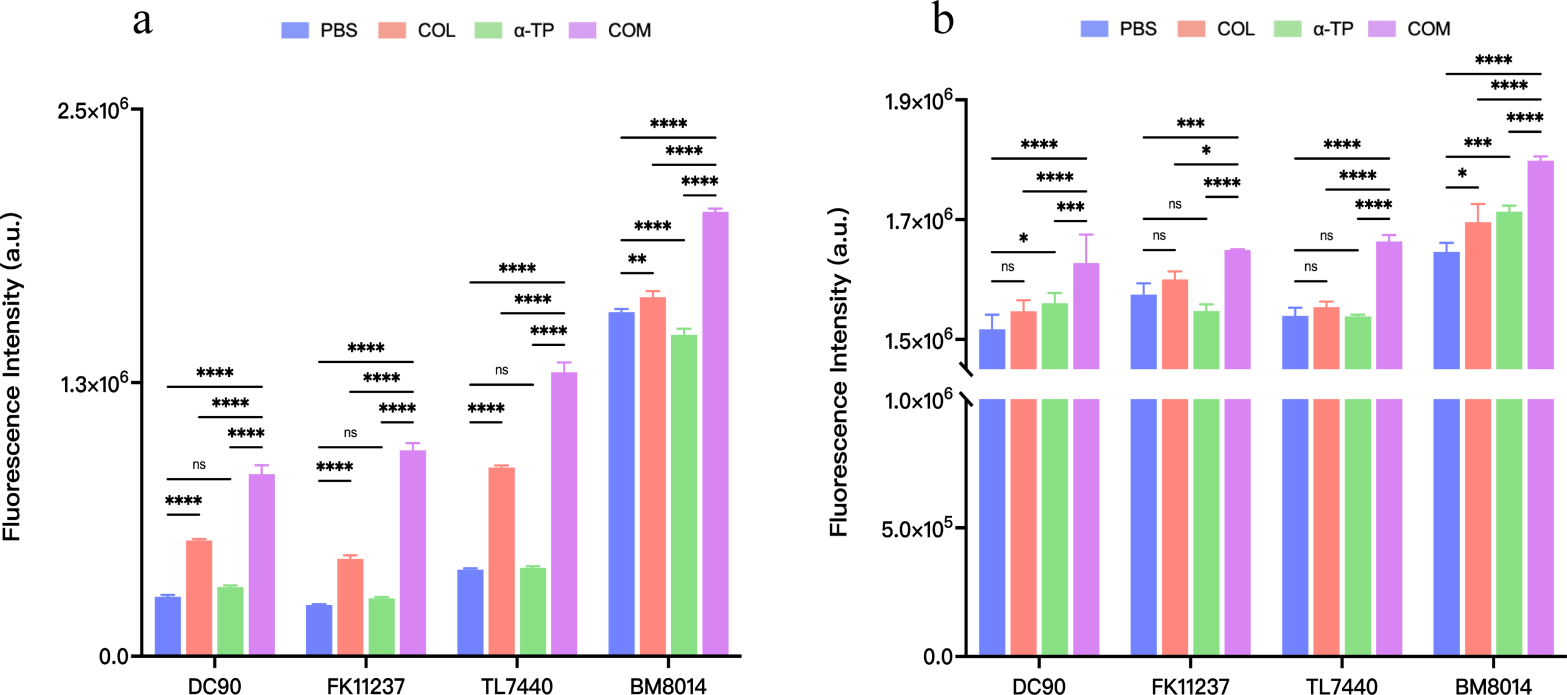
Fluorescence intensity values of DC90, FK11237, TL7440, and BM8014 after different treatments and incubation with (**a**) NPN and (**b**) PI. COM, combination group; DC, *Escherichia coli*; FK, *Klebsiella pneumoniae*; TL, *Pseudomonas aeruginosa*; and BM, *Acinetobacter baumannii*. *P < 0.05; **P < 0.01; ***P < 0.001; ****P < 0.0001; and ns, *P* > 0.05.

The effectiveness of COL combined with α-TP against COL-R GNB may be attributable to mechanisms that increase harmful levels of reactive oxygen species (ROS), destroy bacterial cell membranes, and have a broad-spectrum antibacterial effect. Four strains (DC90, FK11237, TL7440, and BM8014) were randomly selected from the 32 strains for fluorescence quantification of ROS, superoxide anions, and bacterial outer and inner membrane permeability. The results of ROS and superoxide anions are shown in Fig. 8. Fluorescence intensity of bacteria was substantially increased after the COL and α-TP combined treatment. This suggested that the combined treatment may have led to ROS accumulation within bacteria, thereby promoting oxidative stress. In Fig. 9, fluorescent dye N-phenyl-1-naphthylamine (NPN) and propidium iodide (PI) dye were used to evaluate bacterial outer membrane permeability and inner membrane permeability, respectively. Fluorescence levels were significantly higher in the combined treatment group, implying that the combined treatment successfully increased the permeability of the outer and inner bacterial membranes. In conclusion, COL combined with α-TP may have had a synergistic antimicrobial effect through ROS accumulation and increased permeability of bacterial outer and inner membranes.

### Scanning electron microscopy results

Scanning electron microscopy (SEM) was used to investigate the synergistic antibacterial action of COL combined with α-TP on bacterial cell membranes (Fig. S1). FK12716 was used in these experiments. The results demonstrated that bacteria were densely distributed in both the single-drug and control groups (COL: 1 µg/mL and α-TP: 128 µg/mL). Bacterial cell membranes were intact and smooth, and no scar formation was observed; however, when COL and α-TP were combined, bacterial numbers were significantly reduced. Damage to bacterial cell membranes and scar formation was observed (10,000×).

### Antimicrobial activity *in vivo*

**Fig 10:**
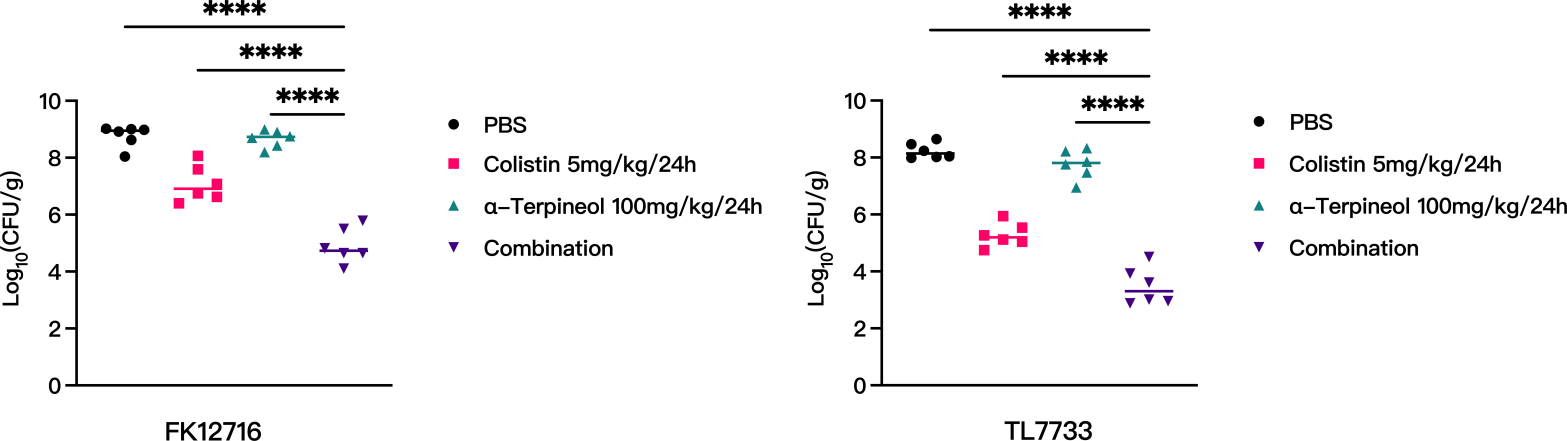
Quantitative analysis of bacterial load in the thighs of mice infected with FK12716 and TL7733 treated with COL and α-TP alone and in combination after 24 h (Log_10_ CFU/g). ****P < 0.0001.

Antimicrobial activity of the combined therapy *in vivo* was evaluated using a mouse thigh infection model. Mouse thigh infection models were constructed using FK12716 and TL7733. After 2 h of infection, mice were treated using monotherapy (COL [5 mg/kg of body weight] or α-TP [100 mg/kg of body weight]) or combination therapy (COL [5 mg/kg of body weight] + α-TP [100 mg/kg of body weight]). The results showed that COL combined with α-TP had stronger antimicrobial activity than either COL or α-TP alone (*P* < 0.05) (Fig. 10). This also indicated that COL combined with α-TP had a significant synergistic antimicrobial effect *in vivo*.

## DISCUSSION

In 2024, the World Health Organization listed 24 pathogens belonging to 15 families of antibiotic-resistant bacterial pathogens, which represent serious human health threats, with a majority classified as GNB. COL is a cationic lipopeptide antibiotic that exhibits both nephrotoxic and neurotoxic effects (14). The primary reason for limiting the COL dose is nephrotoxicity (15). Consequently, clinical researchers are striving to identify effective strategies to lower the COL dosage while enhancing its therapeutic efficacy and minimizing toxicity. For example, the combination of COL and rifampicin in an aerosolized powder for dual administration reduced drug resistance and toxicity while achieving a synergistic antibacterial effect (16). Furthermore, the combination of COL with antioxidants such as melatonin reduces toxicity to the kidney. Clinical evaluations indicate an increasing rate of COL resistance among MDR GNB, making it impractical to rely solely on COL or to increase its dosage. Thus, combination therapy may be a pivotal strategy for addressing this challenge (17). Combination therapy has become popular in the medical field due to its positive results (18). Combinations of COL with other antimicrobial and nonantimicrobial drugs have been reported (19–22).

Natural plant extracts can be used as adjuvants for antibacterial or antifungal drugs to enhance their antibacterial and antifungal abilities (23). For example, nimbolide extracted from neem trees has an obvious antibacterial effect on *Helicobacter pylori in vitro* (24), and dehydrolactone extracted from fresh plant stems has the potential to act as an antibacterial adjuvant against methicillin-resistance *Staphylococcus aureus* (25). In addition, resveratrol, a commercial plant extract, has significant antibacterial effects on *E. coli* and *S. aureus* (26). The use of plant extracts as antibacterial adjuvants is highly promising because they are affordable, secure, and easily obtained. α-TP is a representative terpenoid found in plant essential oils. It is derived from turpentine and is extensively used in everyday formulations and edible flavoring. α-TP has anti-inflammatory effects that protect against dextran sodium sulfate-induced colitis by lowering inflammatory and apoptotic responses; it also reduces inflammation in carrageenan-induced pleurisy and has shown potential as a treatment for various neurodegenerative illnesses (10, 27–29). Research shows that α-TP combined with gentamicin has significant antitoxic and antibacterial action against *Pseudomonas aeruginosa* (30). Furthermore, α-TP affects membrane permeability (31). COL interacts electrostatically with the negatively charged lipid A on bacterial LPS, causing depolarization and breakdown of the outer membrane of GNB, eventually leading to cell death by osmotic lysis. Therefore, in this study, α-TP was selected as the primary candidate based on the following considerations: first, this extract is widely available in nature, which greatly facilitates its potential clinical translation in the future. Second, in the pharmaceutical field, α-TP has been identified as possessing anti-inflammatory, antitumor, and other beneficial properties, demonstrating significant pharmacological value. Third, previous studies have already investigated α-TP in mouse/rat models, indicating its potential for *in vivo* applications. In future studies, we will endeavor to identify α-TP with potential daily-life applications and investigate its capacity to restore COL susceptibility in COL-R bacterial strains.

In the present study, we aimed to investigate the combined antimicrobial effects of COL and α-TP against COL-R GNB in a novel manner. Most of the strains selected for this study were MDR. The combination of COL and α-TP notably diminished the MICs across all tested strains, ranging from 4- to 2,048-fold, suggesting that this combination enhanced the antimicrobial efficacy of COL. In addition to COL-R strains, we also examined eight COL-S bacterial isolates. According to the experimental results (Table S2), the combination of COL and α-TP showed excellent synergistic antibacterial effects against COL-S strains (FICI < 0.5). Therefore, this combination therapy not only effectively combats COL-R strains but also demonstrates good efficacy against COL-S strains, indicating potential clinical application value. Time-kill assays revealed shifts in bacterial populations over 24 h post-treatment with COL alone, α-TP alone, and the combination treatment. Within the initial 2 h, the bacterial counts of all strains began to decline following treatment with the combination. Notably, complete eradication was not achieved for most bacterial strains, even at 2× FICI combination concentrations. We propose two potential explanations for this observation. First, although the drug combination demonstrated significant antibacterial effects during 4–12 h of treatment, its efficacy gradually diminished with prolonged exposure due to decreasing drug concentrations. Second, during the experimental process, some drug-resistant strains formed structurally dense biofilms on the culture tube walls. This growth state substantially enhances bacterial tolerance to antimicrobial agents. Consequently, nearly all tested strains exhibited regrowth after extended exposure periods. But the drug concentrations used in the time-kill assay were significantly lower than those employed in the mouse infection model. Therefore, the *in vitro* time-kill results alone cannot fully reflect the therapeutic efficacy *in vivo*. With more refined studies on dosing regimens and host factors, the combination therapy may demonstrate even stronger antibacterial effects. Second, the time-kill results revealed excellent synergistic activity between the 4 and 12-h treatment. This suggests that intermittent dosing (e.g., twice or multiple times) could be considered in *in vivo* treatments to sustain antimicrobial efficacy. In summary, although this combination therapy has not yet been tested in humans, our murine experiments provide compelling evidence for its potential therapeutic utility *in vivo*.

Bacterial cells in biofilms are more resistant to these antimicrobial agents than those in the planktonic phase (32). Biofilms pose considerable safety hazards in clinical and hospital environments, leading to recurrent chronic and nosocomial infections. Therefore, it was essential to explore whether the combination therapy used in this study could effectively inhibit bacterial biofilms. The findings indicated that combination therapy has the potential to decrease mortality rates and the incidence of hospital-acquired infections. The crystal violet assay demonstrated that the combined therapy effectively reduced biofilms; however, viable bacterial counts revealed differential effects among the tested strains. In the biofilm inhibition assay, seven out of eight strains showed significant reductions in viable counts after combination treatment; in established biofilms, six out of eight strains exhibited marked decreases in viability. We hypothesized that this discrepancy may have stemmed from the particularly robust biofilm-forming capacity of the two tested *P. aeruginosa* strains. The combination treatment may have partially degraded extracellular polymeric substances without achieving complete penetration or bactericidal effects within the mature biofilm architectures. This is also a limitation of our current study, and we hope to further address this issue through continuous exploration in future research.

Cells consistently generate ROS during aerobic respiration, whereas antioxidant defenses mitigate excessive ROS production. Oxidative stress occurs when ROS generation surpasses the endogenous antioxidant capacity of cells (33). After the combination treatment, the bacterial strains examined in the present study exhibited a noticeable increase in ROS levels. Superoxide anion, recognized as one of the most critical ROS, was quantitatively measured, and bacterial samples subjected to combination therapy showed significantly elevated superoxide anion production. It should be noted that our analysis did not include other ROS components such as hydrogen peroxide or hydroxyl radicals. Therefore, the potential upregulation of these additional oxidative species following the combination treatment cannot be ruled out and warrants further investigation in subsequent studies to comprehensively elucidate this aspect. NPN and PI test results demonstrated that the combination treatment increased the permeability of the inner and outer bacterial membranes. The bacterial outer membrane serves as an essential cellular component that functions as a selective permeability barrier, protecting against harmful external substances, and represents the primary target disrupted by combination drug therapy. Therefore, we conclude that outer membrane disruption was a critical antimicrobial mechanism of this combination therapy. The synergy of α-TP and COL may have undermined bacterial cell membrane integrity, resulting in bacterial rupture, as supported by SEM results. This finding is similar to those of previous studies (13).

α-TP was shown to be safe both *in vivo* and *in vitro*, with no cytotoxicity shown in murine macrophages at a concentration of 500 µg/mL. Furthermore, the 50% oral lethal dose for both rats and mice was >5,000 mg/kg of body weight (9, 34). The present study used cytotoxicity and erythrocyte hemolysis assays to show that α-TP, either alone or in combination with COL, was safe *in vitro*. Through H&E staining of tissue organs from treated mice, we could preliminarily conclude that the experimental concentration demonstrated *in vivo* safety. We used mouse thigh infection models to test the antibacterial effectiveness of the α-TP and COL combination *in vivo*. The combination therapy significantly decreased the bacterial burden in the thighs of mice according to the experimental data, indicating possible therapeutic effects *in vivo*. Notably, prior research has clearly characterized the pharmacokinetics of α-TP (31) and demonstrated that α-TP exhibits excellent oral bioavailability *in vivo*, with efficient absorption and distribution. Particularly noteworthy was its high absorption efficiency in the gastrointestinal tract. In our subsequent investigations, we intend to further optimize both the dosage regimens and administration protocols.

In conclusion, this study examined the antimicrobial activity of α-TP in combination with COL against COL-R GNB both *in vitro* and *in vivo*, offering a possible therapeutic option to address the COL resistance issue that is becoming increasingly of clinical concern.

## MATERIALS AND METHODS

### Bacterial isolates and growth conditions

Thirty-two COL-R isolates of COL-R GNB were randomly selected from the First Hospital of Wenzhou Medical University, China. These strains were stored at −80°C in Luria‒Bertani (LB) broth containing 30% glycerol and subjected to further experiments.

### Antibiotics and chemicals

α-TP was purchased from MedChemExpress Co., Ltd., NJ, USA, and dissolved in dimethyl sulfoxide (5%, vol/vol) (Sigma-Aldrich, St. Louis, MO, USA). Colistin, ciprofloxacin, aztreonam, cefepime, imipenem, gentamicin, levofloxacin, and tobramycin were purchased from Wenzhou Kangtai Biotechnology Co., Ltd.

### Antimicrobial susceptibility testing

MICs of all antibiotics and α-TP against 32 COL-R GNB strains were tested using the broth microdilution method (35). Bacterial suspensions and compounds were mixed in the plates and incubated for 16–18 h at 37°C. Antimicrobial susceptibility test results were evaluated using CLSI 2023 guidelines.

### Checkerboard assays

The antimicrobial effects of this combination *in vitro* were assessed using checkerboard assays (36). Briefly, a dilution series of COL and α-TP concentrations was added to the plates to obtain different dilution combinations. Then, 100 µL of bacteria was added to 96-well plates. FICI values were used to assess antibacterial activity, and they were calculated as FICI = FIC_α-TP_ + FIC_COL_ = (MIC_α-TP combined with COL_/MIC_α-TP alone_) + (MIC_COL combined with α-TP_/MIC_COL alone_). FICI values ≤ 0.5 indicate synergism, those in the range of 0.5–4 indicate no effect, and values >4 indicate antagonism (37).

### Time-kill assays

Time-kill assays were conducted to evaluate the impact of COL combined with α-TP on bacterial growth kinetics, as previously reported (38). Eight strains were randomly selected from the following tested strains: *E. coli*, *K. pneumoniae*, *A.baumannii*, and *P. aeruginosa*. Treatment groups included the phosphate-buffered saline (PBS), COL, α-TP, and combination (COL + α-TP). Concentrations were determined based on the checkerboard assay results, with the selected concentration set at 2× FICI value. Next, 200 µL of 0.5 McFarland standard bacteria was added to 20 mL MH broth containing corresponding compounds, which were cultured at 37°C with shaking at 180 rpm. Colony counts of the four treatment groups were determined at 0, 2, 4, 6, 12, and 24 h, respectively.

### Crystal violet staining assays

Biofilm formation inhibition and mature biofilm removal assays were performed as described previously (39, 40) using eight randomly selected COL-R GNB strains. The compound concentrations were 0.5× FICI for the biofilm formation inhibition assay and 2× FICI for the mature biofilm removal assay. For biofilm inhibition, the bacteria were treated with the compounds during biofilm formation. After 24 h, the plates were washed three times to remove unattached cells, stained with 1% crystal violet (250 µL), and dissolved in 95% ethanol and 5% acetic acid (200 µL). For mature biofilm removal, preformed biofilms were treated with the compounds for 24 h and then processed similarly. Absorbance (OD_595_) was measured using a Multiskan FC microplate reader.

### Count of live bacteria in biofilms

Viable bacterial counts in biofilm assays were determined according to a previously published protocol (41). The selected bacteria and compound concentrations were consistent with those used in the crystal violet assay. Briefly, after incubation (under the same conditions as for the crystal violet assay described above), each plate was washed three times with PBS for 24 h to remove non-adherent bacteria. Equal volumes of PBS were added, followed by vortexing. Serial dilutions were plated onto LB agar to assess the number of CFUs.

### CLSM

Live/dead staining of bacteria in biofilm was performed using PI and SYTO 9 (42). After adjusting the turbidity of FK12716 to that of a 0.5 McFarland standard, the bacterial suspension was added to confocal dishes, followed by treatment with the compounds (0.5× FICI). After 24 h of incubation, planktonic bacteria were removed, and fluorescent dyes were added. Observations were made under fluorescence microscopy (Keyence, China). The *Z*-axis was scanned from the initial fluorescence appearance (top layer) to its disappearance (bottom layer).

### Cell toxicity

Cytotoxicity was detected using a cell counting kit-8 (CCK-8) assay (37, 43). RAW264.7 and HEK-293T cells were treated with 10 µL COL (1, 2, or 4 µg/mL), α-TP (64, 128, 256, or 512 µg/mL), or COL + α-TP for 16 h. Subsequently, 10 µL of CCK-8 reagent was added, and the mixture was incubated for 1 h in the dark. A microplate reader (Multiskan FC) was used to measure the absorbance of each well at 450 nm (OD_450_).

### Hemolysis assay

Venous blood from mice was used to assess erythrocyte hemolysis (44). Treatment groups included a negative control (PBS), positive control (0.1% Triton X-100 solution), COL (1, 2, or 4 µg/mL), α-TP (64, 128, 256, 512, or 1,024 µg/mL), and combination (COL + α-TP) groups. The compounds were mixed with erythrocytes and incubated for 2 h and then centrifuged at 3,000 rpm to obtain the supernatant. Using a microplate reader (Multiskan FC) to measure absorbance at 545 nm, the hemolysis rate of red blood cells was then calculated (45).

### Histological sections of mouse visceral tissues

Mice were divided into four groups and intraperitoneally injected with PBS, COL (5 mg/kg of body weight), α-TP (100 mg/kg of body weight), or COL + α-TP. After seven consecutive days of administration, the mice were euthanized. The major visceral organs, including the heart, liver, spleen, lungs, and kidneys, were collected for histological sectioning and HE staining (46). The sections were examined under microscopy (Keyence, China).

### Detection of ROS production

A previously reported protocol for detecting ROS was followed (47). Four strains (DC90, FK11237, TL7440, and BM8014) were randomly selected from the COL-R GNB. The compound concentration was set at 0.5× FICI value. Bacterial suspensions were adjusted to OD_600_ = 0.3–0.4 and then mixed with the fluorescent probe diacetyldichlorofluorescein. Compounds were added after the probe was loaded and incubated for 2 h. Fluorescence intensity was immediately detected using an enzyme labeler (BioTek, Synergy) with excitation at 488 nm and emission at 535 nm.

### Superoxide anion assay

Superoxide anion levels were assessed using an ROS Assay Kit for Superoxide Anion with Dihydroethidium (DHE) (Beyotime Biotechnology Co.). Changes in superoxide anions in bacteria were determined according to the manufacturer’s instructions. The concentrations of selected bacterial strains and compounds were consistent with those used for the ROS assay. Compounds were added after the bacterial suspensions were adjusted to OD_600_ = 0.3–0.4 and incubated for 2 h. The supernatant was then discarded, and DHE dye was added, followed by incubation at 37°C for 30 min. Finally, fluorescence intensity was measured using an enzyme labeler (BioTek, Synergy) with excitation at 535 nm and emission at 610 nm.

### Outer membrane permeability

Extracellular membrane permeability was measured using NPN (Aladdin, Shanghai, China) (48). Four strains (DC90, FK11237, TL7440, and BM8014) were randomly selected from the COL-R GNB. The compound concentration was 0.5× FICI value. Bacterial suspensions were adjusted to OD_600_ = 0.3–0.4. After incubating the bacteria with the compounds for 2 h, they were centrifuged at 4,000 rpm for 5 min and washed three times with PBS. Then, the bacteria were treated with 30 µg/mL of NPN for 30 min. They were then detected using an enzyme marker (Aladdin, Shanghai, China) with excitation at 350 nm and emission at 420 nm.

### Inner membrane permeability

Inner membrane permeability was measured using PI (Solarbio, Beijing, China) (49). Four strains (DC90, FK11237, TL7440, and BM8014) were randomly selected from the COL-R GNB. The compound concentration was 0.5× FICI value. Bacterial suspensions were adjusted to OD_600_ = 0.3–0.4, and the compounds were added and incubated for 2 h. Following a 5-min centrifugation at 4,000 rpm and washing three times with PBS, the bacteria were treated with PI (50 µg/mL) for 30 min. Bacteria were identified using an enzyme marker (Aladdin, Shanghai, China) with excitation at 535 nm and emission at 615 nm.

### SEM

With a few small modifications, SEM was performed in accordance with an established protocol (50). FK12716 morphology and cell membrane integrity were analyzed using SEM. A total of four treatment groups were divided into control (PBS), COL (1 µg/mL), α-TP (128 µg/mL), and combination (COL + α-TP) groups. The bacteria mixed with the compounds were incubated and centrifuged to collect the bacteria, which were then washed twice with PBS. Five hundred microliters of 2.5% glutaraldehyde was added, and the bacteria were fixed for 6 h. To ensure maximum cleanliness, the bacteria were washed twice with PBS and treated with glutaraldehyde for 2 h. They were then dehydrated in a series of ethanol dilutions (30%, 50%, 70%, 80%, 90%, and 100% [vol/vol], 15 min each). Finally, anhydrous ethanol (100 µL) was added for dispersion, dropped onto clean plates, and dried overnight at 37°C in an incubator. Gold was sputtered for 90 s and observed under SEM.

### *In-vivo* analyses of synergistic antibacterial effectiveness

The efficacy of combined α-TP and COL was assessed using an adapted form of a previously documented murine thigh infection model (51). Five- to six-week-old male BALB/c mice weighing 25 g (Charles River, Hangzhou, China) were used in this experiment. National guidelines for animal care and ethics were followed for all animal tests, and any surviving mice were euthanized at the conclusion of the study. To create neutropenic mice, cyclophosphamide (150 mg/kg of body weight) was administered intraperitoneally for three consecutive days. Four groups were used: PBS, COL, α-TP, and combination groups, with three mice in each group. All mice were injected with 100 µL bacterial solution into the posterior thigh muscle, using COL-R strains (FK12716 and TL7733). After 2 h, mice were intraperitoneally injected with 200 µL of 5 mg COL/kg of body weight, 100 mg α-TP/kg of body weight, or 5 mg COL/kg of body weight + 100 mg α-TP/kg of body weight. Within 24 h of treatment, the thighs of all mice were removed, and CFU counts were used to measure the bacterial burden.

### Statistical analysis

GraphPad Prism 9.0 (GraphPad Software, LLC; San Diego, CA, USA) was used for statistical analysis. Data are presented as mean values ± standard deviations from three or more replicate trials. To examine the experimental results, Student’s *t*-test and one-way analysis of variance were used. For all analyses, *P < 0.05; **P < 0.01; ***P < 0.001; ****P < 0.0001; and ns, *P* > 0.05.

## Data Availability

All original data generated during the study are included in the article in the form of figures and tables. For further inquiries, please contact the corresponding author.

## SUPPLEMENTAL MATERIAL

Supplemental material spectrum.00825-25-s0001.docx Table S1 and S2; Fig. S1.

ASM does not own the copyrights to Supplemental Material that may be linked to, or accessed through, an article. The authors have granted ASM a non-exclusive, world-wide license to publish the Supplemental Material files. Please contact the corresponding author directly for reuse.
