# Supplementary material for: Restoring colistin sensitivity in colistin-resistant gram-negative bacteria: combinatorial use of α-terpineol and colistin
Source: Microbiol Spectr. 2025 Sep 30;13(11):e00825-25. doi: 10.1128/spectrum.00825-25 (PMC12584673; doi:10.1128/spectrum.00825-25)
Supplement: Supplemental material — Table S1 and S2; Fig. S1. [file spectrum.00825-25-s0001.docx]

**Table S1.** The MICs of commonly used clinical antibiotics and α-terpineol against Col-R GNB

| **Species** | **Strains^a^** | **Antibiotics^b^** | | | | | | | | **α-Terpineol** |
| --- | --- | --- | --- | --- | --- | --- | --- | --- | --- | --- |
|  |  | **Breakpoints (S-R)^c^ MIC (mg/L)** | | | | | | | |  |
|  |  | **ATM** | **FEP** | **IPM** | **CIP** | **LVX** | **GEN** | **TOB** | **COL** |  |
|  |  | **4-16** | **2-16** | **1-4** | **0.25-1** | **0.5-2** | **4-16** | **4-16** | **2-4** |  |
| *E. coli* | **DC90** | **≥256** | **64** | **≥256** | **64** | **32** | **≥256** | **128** | **4** | 1024 |
|  | **DC3599** | **64** | 8 | 1 | **4** | **8** | **16** | 8 | **8** | 1024 |
|  | **DC3737** | **≥256** | **≥256** | **128** | **≥256** | **≥256** | **≥256** | **≥256** | **4** | 1024 |
|  | **DC3846** | **128** | **≥256** | 0.5 | **≥256** | **128** | **≥256** | **64** | **4** | 1024 |
|  | **DC5286** | **≥256** | **≥256** | 0.25 | **128** | **64** | 4 | 4 | **4** | 1024 |
|  | DC19144 | ≤1 | ≤1 | ≤1 | **≥4** | **≥8** | ≤1 | ≤1 | **8** | 1024 |
|  | DC19526 | ≤1 | ≤1 | ≤1 | **≥4** | **≥8** | ≤1 | ≤1 | **4** | 1024 |
|  | **DC19829** | ≤1 | ≤1 | ≤1 | **≥4** | **≥8** | **≥16** | 8 | **8** | 1024 |
|  |  |  |  |  |  |  |  |  |  |  |
| *K. pneumoniae* | **FK169** | 1 | 0.5 | **4** | **2** | 1 | 1 | **64** | **≥256** | 1024 |
|  | **FK1913** | **≥128** | **≥128** | **32** | **≥128** | **≥128** | **≥128** | **≥128** | **≥256** | ≥2048 |
|  | **FK3994** | **≥256** | **≥256** | **32** | **≥256** | **64** | **≥256** | **≥256** | **64** | 1024 |
|  | **FK6556** | **64** | **64** | **16** | **4** | **8** | **16** | **16** | **8** | 1024 |
|  | **FK6663** | **≥256** | **≥256** | **32** | **≥256** | **≥256** | **≥256** | **≥256** | **4** | ≥2048 |
|  | **FK6696** | **≥256** | **≥256** | **128** | **≥256** | **64** | **≥256** | **≥256** | **128** | 1024 |
|  | FK11237 | ≤1 | ≤1 | **≥16** | ≤0.25 | ≤0.25 | ≤1 | ≤1 | **4** | 1024 |
|  | **FK12771** | **≥64** | **≥64** | **≥16** | **≥4** | **≥8** | **≥16** | **≥16** | **64** | 1024 |
|  | FK12716 | ≤1 | ≤1 | ≤1 | ≤0.25 | ≤0.25 | ≤1 | ≤1 | **16** | 1024 |
|  |  | **-** | **8-32** | **2-8** | **1-4** | **2-8** | **4-16** | **4-16** | **2-4** |  |
| *A. baumannii* | **BM1342** | 64 | **≥256** | ≤0.25 | **64** | **32** | 1 | 1 | **4** | 1024 |
|  | **BM1412** | 128 | **≥256** | **8** | **256** | **32** | 8 | 1 | **4** | 1024 |
|  | **BM2431** | 64 | **64** | **16** | **4** | **8** | 1 | 1 | **4** | 1024 |
|  | **BM7477** | 64 | **≥64** | **≥16** | **≥4** | 4 | **≥16** | **≥256** | **128** | 1024 |
|  | **BM7970** | 64 | **≥64** | **≥16** | **≥4** | **≥8** | **≥16** | **≥16** | **128** | 512 |
|  | **BM7994** | 32 | **≥64** | **≥16** | **≥4** | 4 | **≥16** | **≥16** | **16** | 1024 |
|  | **BM8014** | 16 | ≥8 | **16** | **128** | **≥16** | **≥256** | **≥256** | **16** | 1024 |
|  |  | **8-32** | **8-32** | **2-8** | **0.5-2** | **1-4** | **4-16** | **4-16** | **2-4** |  |
| *P. aeruginosa* | TL1671 | 8 | 8 | 2 | 0.25 | 1 | 2 | 1 | **4** | ≥2048 |
|  | **TL7333** | 16 | **64** | 2 | **2** | **≥8** | **≥16** | **64** | **16** | ≥2048 |
|  | **TL7440** | ≥16 | **64** | **≥8** | **≥4** | **≥4** | **≥16** | 0.5 | **16** | ≥2048 |
|  | TL7505 | 4 | ≤0.25 | ≤1 | **≥4** | 2 | 2 | 0.5 | **8** | ≥2048 |
|  | TL7548 | 4 | **32** | 2 | 0.5 | 0.5 | 2 | ≤1 | **16** | ≥2048 |
|  | **TL7733** | **32** | **64** | 2 | 1 | 2 | ≤1 | ≤1 | **8** | ≥2048 |
|  | **TL7929** | 4 | **256** | 4 | **≥8** | ≤0.25 | 2 | 1 | **32** | ≥2048 |
|  | **TL8126** | 4 | **256** | **≥16** | 0.5 | 0.5 | ≤1 | ≤1 | **32** | 1024 |

^a^strains in boldface are multidrug-resistant (MDR) strains.

^b^ATM, aztreonam; FEP, cefepime; IPM, imipenem; CIP, ciprofloxacin; LVX, levofloxacin; GEN, gentamicin; TOB, tobramycin; COL, colistin.

^c^S-R represents the susceptible (S) breakpoint to resistant (R) breakpoint, according to CLSI supplement M100 (30th edition) and EUCAST.

**Table S2.** FICI values for colistin/α-terpineol combinations against COL-S GNB

| **Species** | **Strain** | **Monotherapy MIC (μg/mL)** | | **Combination MIC (μg/mL)** | | **FICI** | **Interpretation** |
| --- | --- | --- | --- | --- | --- | --- | --- |
|  |  | **Colistin** | **α-Terpineol** | **Colistin** | **α-Terpineol** |  |  |
| *E. coli* | DC11723 | 0.25 | ≥1,024 | 0.03125 | 64 | ≤0.1875 | Synergistic |
|  | DC12843 | 0.25 | ≥1,024 | 0.03125 | 128 | ≤0.25 | Synergistic |
| *K. pneumoniae* | FK8160 | 0.125 | ≥1,024 | 0.015625 | 64 | ≤0.1875 | Synergistic |
|  | FK8165 | 0.125 | ≥1,024 | 0.015625 | 64 | ≤0.1875 | Synergistic |
| *A. baumannii* | BM6913 | 0.5 | ≥1,024 | 0.03125 | 256 | ≤0.3125 | Synergistic |
|  | BM6927 | 0.5 | ≥1,024 | 0.125 | 128 | ≤0.375 | Synergistic |
| *P. aeruginosa* | TL7559 | 1 | ≥1,024 | 0.25 | 16 | ≤0.265625 | Synergistic |
|  | TL7553 | 0.5 | ≥1,024 | 0.0625 | 128 | ≤0.25 | Synergistic |


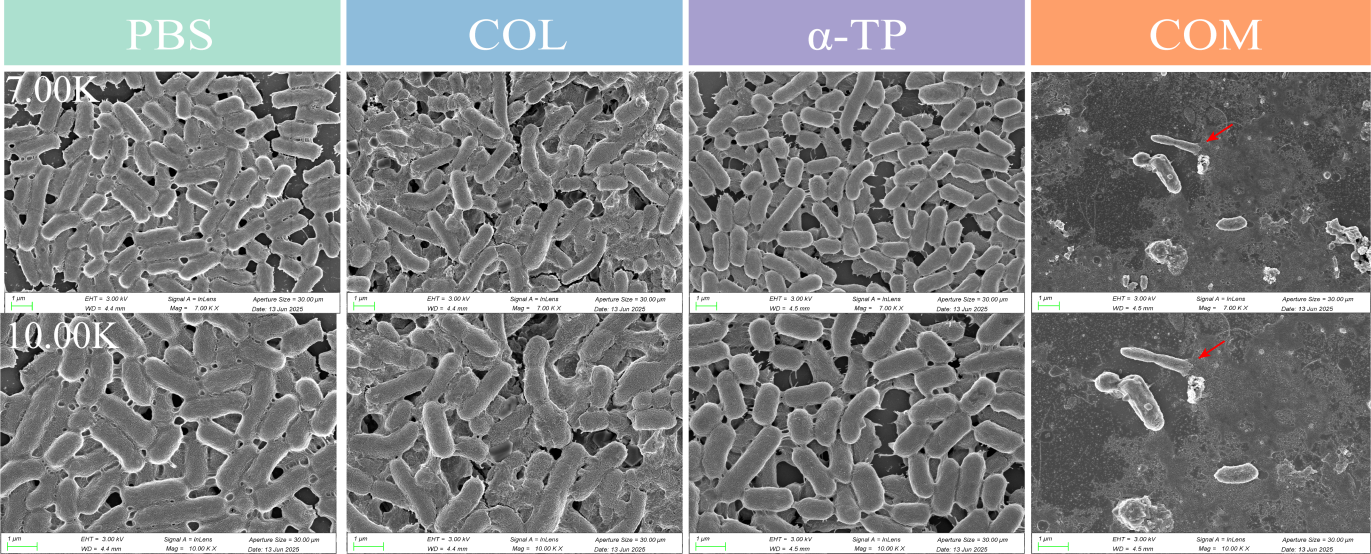


**Figure S1** Bacterial cell membrane disrupting effects of COL, α-TP and combined groups on FK12716. COM: combination group. FK stands for *K.pneumoniae*.
